# Motivation and challenges for use of malaria rapid diagnostic tests among informal providers in Myanmar: a qualitative study

**DOI:** 10.1186/s12936-015-0585-7

**Published:** 2015-02-06

**Authors:** May Sudhinaraset, Christina Briegleb, Moe Aung, Hnin Su Su Khin, Tin Aung

**Affiliations:** Department of Epidemiology and Biostatistics, University of California, San Francisco, CA USA; Global Health Group, Global Health Sciences, University of California, San Francisco, CA USA; Population Services International, Yangon, Myanmar

**Keywords:** Myanmar, Malaria, Rapid diagnostic tests, Private healthcare provider, Informal provider, Qualitative

## Abstract

**Background:**

Rapid diagnostic tests (RDTs) for malaria enable proper diagnosis and have been shown to reduce overuse of artemisinin combination therapy. Few studies have evaluated the feasibility and use of RDTs in the private sector in Myanmar. The objectives of the study were to: 1) understand the acceptability of using RDTs in the informal sector in Myanmar; 2) examine motivations for use among informal providers; and, 3) highlight decision-making and knowledge of providers for diagnostic testing and treatment.

**Methods:**

Qualitative interviews were conducted with 30 informal providers. Purposeful sampling was used to enrol study participants in the Mon and Shan State in Myanmar. All interviews were conducted in Burmese, translated into English, and two researchers coded all interviews using Atlas ti.

**Results:**

Major themes identified included: 1) informal provider and outlet characteristics, including demographic and background characteristics; 2) the benefits and challenges of using RDTs according to providers; 3) provider experiences with using RDTs, including motivations for using the RDT; 4) adherence to test results, either positive or negative; and, 5) recommendations from informal providers to promote increased use of RDTs in their communities. This study found that introducing RDTs to informal providers in Myanmar was feasible, resulting in improved provider empowerment and patient-provider relationships. Specific challenges included facility infrastructure to use and dispose RDTs and provider knowledge. This varied across the type of informal provider, with itinerant drug vendors more comfortable and knowledgeable about RDTs compared to general retail sellers and medical drug representatives.

**Conclusions:**

This study found informal providers in Myanmar found the introduction of RDTs to be highly acceptable. Providers discussed improvement in service quality including provider empowerment and patient-provider relationships. The study also highlighted a number of challenges that informal providers face which may be used for future development of interventions.

## Background

Anti-malarial drug resistance is a significant threat in global efforts to control malaria. Myanmar in particular recently reported the highest mortality due to malaria in the Greater Mekong Sub-Region [1]. Alarming recent reports indicate emergence of artemisinin resistance *Plasmodium falciparum* in the eastern region, threatening current efforts and improvements in malaria control and elimination programmes [2]. Presumptive treatment with the first-line drug, artemisinin-based combination therapy (ACT), for suspected rather than confirmed malaria cases, may further lead to drug resistance. Rapid diagnostic tests (RDTs) for malaria enable proper diagnosis and have been shown to reduce overuse of ACT [3-7]. Between 2010 and 2011 the Myanmar Artemisinin Resistance Containment (MARC) was developed, with one of the aims to strengthen and improve access to and use of early diagnosis and quality treatment [8]. National rapid scale-up of RDTs is anticipated for 2014-2015, and yet in Myanmar there is a dearth of information on RDT use among healthcare providers. Given that only 33% of the Myanmar population lives in urban areas [9], understanding RDT use in rural and remote villages is especially important.

Studies suggest that to maximize the potential of RDTs, private informal providers play a significant role in malaria diagnosis and treatment [10-12]. Informal private providers are a part of the private sector, but typically lack formal training and oversight. In Uganda, two-thirds of medicines are delivered through the private sector, mostly from drug shops [11]. The private sector is critical in Myanmar, with 80% of fever patients seeking care in retail private sector, typically the informal sector, compared to only 11% going to the public sector [13]. Although previous studies demonstrate that communities perceive drug shops as important in treating malaria, communities were also concerned that drug shops were motivated by profits and that they would raise the price of RDTs [12]. A number of studies in Africa and Asia suggest that community health workers are able to prepare and interpret malaria RDTs correctly and safely when supported by appropriate training and clear instructions [14-16].

A few studies evaluating RDTs use and feasibility exist in Myanmar [13,17-19]. Two studies found that introduction of RDTs to relatively inexperienced providers, including village volunteers and village health workers, was feasible [17,19]; however, a number of challenges arose, including insufficient instructions to discriminate mixed *P. falciparum*/*Plasmodium vivax* from *P. falciparum* infections [19] and findings that the number of patients tested declined over time [17]. These studies suggest that continued training and supervision of volunteers are critical for sustaining efforts, but are also limited by their focus only on village health workers or volunteers. Few studies exist on acceptability, motivations and challenges of rolling out RDTs in the informal sector in Myanmar, and importantly, whether different types of informal providers may be most adequate in diagnosing and treating malaria. Understanding these issues will be especially important given the anticipated national roll-out of RDTs.

This study uses qualitative data from in-depth interviews with three types of informal providers in rural Myanmar. The objectives of the study were to: 1) understand the acceptability of using RDTs in the informal sector in Myanmar; 2) examine motivations for use among informal providers; and, 3) highlight decision-making and knowledge of providers for diagnostic testing and treatment.

## Methods

### Study context

This study was the qualitative component of a larger community randomized, controlled trial to evaluate an intervention to improve uptake of RDTs among informal providers in Myanmar. A description of the pilot intervention and methodology are described elsewhere [20]. Briefly, Population Services International/Myanmar (PSI/M) rolled out the pilot intervention in rural Mon and Shan State in Eastern Myanmar between April and September 2013 to approximately 600 private providers. The intervention specifically targeted the informal sector among providers with little or no experience of diagnostic testing, including grocery retail sellers, itinerant drug vendors and medical drug representatives. General retail sellers own small shops in the community and typically sell a variety of goods, including non-health related products. Itinerant drug vendors often operate from their homes and sometimes serve as the village doctor, with some vendors travelling to patients’ homes for care ranging from maternal and child health services to malaria diagnosis and treatment. Medical drug representatives are similar to pharmacists and have knowledge about basic drugs and treatments.

The three intervention arms included: 1) subsidies for RDTs; 2) subsidies and one extra RDT for every five purchased by providers; and, 3) subsidies and information, education and counselling. The qualitative component of the study was carried out in August 2013 among informal providers who were enrolled in the pilot intervention.

### Description of RDT

The study used FIRST RESPONSE^R^ Malaria antigen pLDH/HRP2 combo card test, manufactured by Premier Medical Corp Ltd, Gujarat, India. The device reports three potential results, indicated by three lines on the test: one result for a control (no malaria), one for *P. falciparum*, and one for *P. vivax*.

### Sample and data collection

In total 30 informal providers were included in the qualitative study. Purposeful sampling of informal providers was used for enrolment. Participants were sampled by provider type (grocery retail sellers, itinerant drug vendors and medical drug representatives), and balanced low/high performing providers. The number of providers was also similarly sampled across the intervention arms of the larger study. PSI/M programme data were used to classify low/high performing providers. Providers who frequently restocked RDTs in their outlets were classified as high performing, and low performers were those who rarely restocked their supplies of RDTs. Inclusion criteria to the study were that informal providers were enrolled in PSI/M’s intervention on RDTs, be at least 18 years of age, and working in Mon or Shan State of Myanmar. Researchers from PSI/M contacted a number of informal providers before the interview to alert them that they may be potentially interviewed.

Four research assistants, bilingual in Burmese and English, participated in a three-day qualitative training and conducted all interviews under the supervision of a field manager and a research coordinator. Interviews were conducted in Burmese. Providers were asked about the services they offered at their outlet, their knowledge on malaria and RDTs, and their experience using the RDTs, including challenges and perceived benefits. All interviews took place in a private location identified by the providers, and most occurred at the provider outlet. Interviews lasted approximately 45 minutes to one hour and were tape-recorded. A demographic form was completed after the completion of the interview.

### Data analysis

All in-depth interviews were transcribed and translated directly into English. Two researchers coded all text using Atlas ti. Thematic analysis was used to address the objectives of the study. First, interview transcripts were reviewed in their entirety to identify major themes and to check the quality of transcriptions and translations. Next, textual information from transcriptions were analysed and codes were developed until saturation was reached. Families of codes were developed based on larger thematic objectives, and information was summarized across provider types. Finally, themes across provider types were compared and contrasted.

### Ethical considerations

Ethical approval was obtained by Population Services International’s Ethical Review Board. Research assistants obtained informed consent from all informal providers prior to conducting the interview.

## Results

This section is organized into the major themes identified from the study. They includes: 1) informal provider and outlet characteristics, including demographic and background characteristics; 2) the benefits and challenges of using RDTs according to providers; 3) provider experiences with using RDTs, including motivations for using the RDT; 4) adherence to test results, either positive or negative; and, 5) recommendations from informal providers to promote increased use of RDTs in their communities.

### Informal provider and outlet characteristics

In total 30 informal providers were interviewed: ten general retail sellers, ten itinerant drug vendors and ten medical drug representatives. All completed demographic forms [see Table 1]. Nearly three-quarters of informal providers had a high school education. Typically informal providers have little to no formal medical training, although medical drug representatives and itinerant drug vendors are more likely to have some training compared to general retail sellers. In this sample, however, all informal providers had some form of medical training, and all had been trained by PSI/M. Two had an apprenticeship and both were medical drug representatives. Seventy per cent of informal providers were female, and the mean age was 41 years old. All but two informal providers were owners of the outlet.

**Table 1:** **Demographic characteristics of providers, n (%)**

**Demographic characteristics**	**Total**	**Medical drug representative (MDR)**	**Itinerant drug vendor (IDV)**	**General retail store (GRS)**
Provider Type	30	10 (33.3)	10 (33.3)	10 (33.3)
Female	21 (70)	7 (70)	7 (70)	7 (70)
Age (mean)	40.5**	41.9	38.3	41.4
Education Level				
Primary	1 (3.3)	0	0	1 (10)
Middle School	2 (6.7)	1 (10)	0	1 (10)
High School	19 (63.3)	5 (50)	7 (70)	7 (70)
University	8 (26.7)	4 (40)	3 (30)	1 (10)
Religion				
Buddhism	26 (86.7)	9 (90)	9 (90)	8 (80)
Christianity	3 (10)	0	1 (10)	2 (20)
Hinduism	1 (3.3)	1 (10)	0	0
Ethnicity				
Bamar	10 (33.3)	4 (40)	3 (30)	3 (30)
Mon	7 (23.3)	3 (30)	3 (30)	1 (10)
Kayin	5 (16.7)	0	1 (10)	4 (40)
Shan	4 (13.3)	2 (20)	1 (10)	1 (10)
Kayar	1 (3.3)	0	1 (10)	0
Indian	1 (3.3)	1 (10)	0	0
Paou	1 (3.3)	0	1 (10)	0
Palaung	1 (3.3)	0	0	1 (10)
Number of fever client/month (mean)	178.6	231	192.8	112
Buying cost of RDT (mean)	107 kyats	105 kyats	120 kyats	95 kyats
Selling cost of RDT (mean)***	278 kyats	295 kyats	390 kyats	150 kyats

**Statistical difference for sex, chi2 p = 0.000.

***Statistical difference for selling cost of RDT, anova p = 0.0340.

Informal providers offered a variety of services at their outlet. All offered malaria services, and 23 offered diarrhoea services. About half of informal providers offered pneumonia and family planning services, and a third pregnancy care, post delivery care, and sexually transmitted infection services; fewer general retail sellers offered these services compared with itinerant drug vendors and medical drug representatives. A small number of informal providers offered delivery (n = 6), tuberculosis (n = 4), and post abortion care (n = 1) services. Three of the six outlets offering delivery were general retail sellers. The mean number of patients presenting with fever per month was 179; medical drug representatives had the most fever patients, while general retail sellers the fewest. All informal providers had RDTs in stock at the time of interview. Supa Arte is an artemisinin-based combination treatment package subsidized by Population Services International. Twenty-four informal providers said Supa Arte was the most common anti-malarial drug they sold, and all but two said Supa Arte was the best drug to treat malaria. All informal providers had ACT in stock at the time of the interview.

### Benefits and challenges

#### Acceptability and benefits of RDTs: “it’s like I have an extra helper”

Overall acceptability of using RDTs was high among informal providers. Almost all providers found that RDTs were easy to use and that it made their work more efficient. A number of informal providers discussed motivations for using RDTs, including provider empowerment and improved provider-patient relationships. Provider empowerment was the most commonly cited reason for using the RDT. For instance, many providers said that it made their jobs “easier” and that they were now capable of diagnosing and treating patients. Informal providers often referred to the RDT as “blood test strip”. Discerning different types of fevers was also important for providers.

*For me we used to be confused about the cause of the disease. We thought it might be a combination of diseases - flu, dengue fever…different fevers have similar symptoms. Flu is associated with fevers, headaches. Typhoid has the same symptoms. Malaria also has the same symptoms. If I suspect that it might be two of these, I can use the test strip to determine which illness it is. It’s good to have the blood test strip*. – General retail seller

*I really need it in order to know whether someone has malaria or not. If I didn’t have it, I wouldn’t know how to treat someone. Using the test strips, I will be able to give better treatment*. – Itinerant drug vendor

Other providers discussed how the RDT greatly improved treatment practices. For example, one provider discussed how s/he no longer needs to guess which medicine to give because of the RDT.

*It’s good if I have the blood test strip because previously I just gave out medicine based on guessing. Now, because I have the test strip, it’s more accurate. I can know the result and if the patient has malaria I can give Supa Arte. If there’s no malaria, I won’t give it to them*. – Medical drug representative

Almost all providers who used RDTs discussed how this increased their confidence in their medical practices.

*It’s like I have an extra helper* [having the RDT]*, who helps me figure out the causes of patient illnesses. It’s not just me trying to figure it out. I’m happy to have the blood test strip.* – Itinerant drug vendor

Improvements in practices also led to increased perceived patient volume and improved patient-provider relationships. A majority of providers said that their patient flow increased after being trained in RDT use and stocking RDTs. A number of informal providers discussed how because they were part of the community, patients preferred to go to their outlets rather than larger hospitals, in part because of cost and transportation issues. A number of providers also discussed that patients had more trust in the providers as a result of the RDT.

*Their* [patients] *perceptions are positive because in the past they have to take the tests on the slides, but now they know the results immediately so they are happy. If they have to go to town to take tests, they will be disappointed. Now they can take the test easily. They trust me even more*. – Itinerant drug vendor

Increased trust and stature in the community also led to increases in patient flow.

*After using the RDTs, patients are more confident in my experience and also my wife’s experience. In addition, my wife and I give the RDT and drugs cheaper compared to other shops. Clients are more likely to recommend others to our shop.* – Medical drug representative

Finally, some informal providers were also motivated by altruistic reasons. Many informal providers had a good understanding of their communities and wanted to improve the health of their communities, not for monetary or personal gains, but because they cared for the people they served.

*For me, profit isn’t my first priority. It* [RDT*] helps me confirm quickly whether people have malaria or not. I wouldn’t say it’s profitable, but it does help me…It helps them* [patients] *a lot.* – Itinerant drug vendor

[When asked why s/he used RDTs]: *I want my village, my community to develop.* – Itinerant drug vendor

A few providers discussed giving the RDT and medications for free, especially for low-income or rural patients.

*I give it free to some patients. For patients who live up on the hill because they are so poor. They are malnourished. I feel sorry for them so I give them free treatment. Free testing. I give drugs for free. It’s people from Lahu village. It’s very far from here. It’s East of here. If they come to me, I don’t charge any money.* – Itinerant drug vendor

#### Provider challenges with RDT use

Informal providers reported few challenges in using RDTs. One challenge reported was having appropriate infrastructure to draw blood. For example, a small number of providers discussed the type of infrastructure needed to dispose of needles adequately, mostly due to fears concerning HIV. Despite using a new needle for every RDT exchange, one provider discussed perceived risks with using needles based on past experiences.

*I have two colleagues that got HIV from used needles…Therefore, I take a lot of care in disposing needles*. – Itinerant drug vendor

*It’s because of the ‘four alphabet disease’* [referring to AIDS]*. They* [patients] *are afraid of taking the test because they are worried that they might get the disease and then won’t know what to do. That’s their thinking and why they are worried to take the test. Only after starting this shop did I start doing the blood test. In my previous shop, I couldn’t do much. I only gave the blood test when their symptoms were really serious.* – Medical drug representative

Other less common challenges a few providers mentioned included quality of the RDTs themselves, including uncertainty with test results when lines either do not appear or appears between two results, not having enough demand from patients (discussed further below), and patients afraid of taking the RDT because of blood pricks. There were some differences in challenges reported by provider type. All itinerant drug vendors were comfortable using RDTs while a few general retail sellers discussed not being qualified to give the RDT because of lack of medical experience.

*The main problem is that I am scared. I’m afraid to use it. I’m not confident in myself. It’s not from the customer side*. – General retail store

### Experiences with use

#### Understanding of the purpose of RDTs

The majority of all three types of informal providers knew that the purpose of the RDT was to detect malaria and that a RDT is needed to confirm malaria.

*The most certain method* [for detecting malaria] *is using blood tests, and it’s* [RDT] *quicker and easier. Otherwise you have to guess based on symptoms*. – Itinerant drug vendor

*It* [RDT] *is useful for malaria. I can know if the patient has which type of malaria.* – Medical drug representative

In addition, nearly all informal providers were also aware that the RDT could distinguish between *P. falciparum vs P. vivax*.

#### Selective use of RDTs for suspected malaria

While informal providers knew the purpose of RDTs, and reported several benefits for using RDTs, this knowledge and perceived benefits did not necessarily translate into universal and consistent use for each patient who presented with fever. Informal providers reported using RDTs only for patients they suspected had malaria, rather than any patient who presented with a fever. The reasons for suspected malaria varied considerably among providers, including suspected symptoms, specific vulnerable populations and seasonality. A large number of informal providers suspected malaria if the patient presented with specific symptoms: fever with chills or sweating or “rigour”, high fever that comes and goes, or prolonged or “regular” fever.

*Some patients have fever with chills and rigor. Some patients have low-grade fever with chills. If the fever is high, the test will show that it’s positive.–* General retail seller

*The fever is regular* [if it is malaria]. *For example if a patient gets a fever during the night time, the next day they’ll get the fever at the same time the next night. Normally you get a fever, sweat, and then the fever goes down. Then the fever comes up again around two or three times a day.* – Itinerant drug vendor

A few informal providers, especially itinerant drug vendors, described treating a fever first with common medicine and using a RDT only if the fever persisted.

*I will give drugs for fever…Then I say if there are symptoms still left on the next day, I will conduct the RDT test*. – Itinerant drug vendor

*If a patient has a high temperature the first day, I give drugs. I give antibiotics and paracetamol. The next day he comes back but still has fever with high temperature and certain kind of vomiting and headache, then I will suspect that he has malaria. Then I will give him the test.* – Itinerant drug vendor

A smaller number of informal providers suspected malaria if the patient had muscle aches, headaches, vomiting, loss of appetite, diarrhoea, or jaundice.

Apart from specific symptoms, other reasons why informal providers suspected malaria were geographic location, profile of the patient, and/or season. Several informal providers discussed that malaria was only common in certain types of patients: migrant or field workers, those who worked on rubber plantations or mines, or those who had travelled north.

[Describing last time used RDT]: *That patient came back from Myawaddy* [border between Myanmar/Thailand]. *They have malaria there. People returning from there typically have malaria.* – Itinerant drug vendor

*Yes people who live here don’t have malaria. I don’t find any malaria from them. I found only one patient who returned from the North region.* – General retail seller

Informal providers also suspected malaria for patients who worked in the field or mines, of which one informal provider also associated with being poor.

*Malaria is common in field workers…most of whom can’t afford* [RDT], *are poor.* – Medical drug representative

[Describing last time used RDT]: *He’s from a gold mine village. Malaria is common in those mining sites.* – General retail seller

*I think it* [malaria] *may be* [present], *especially for people living on the hilly region and also in migrant workers. People on the hills are working in the rubber fields.* – Itinerant drug vendor

Lastly, informal providers also suspected malaria during the rainy season, or for a patient who had malaria previously.

*During the rainy season, malaria is caused by mosquito bites. They have dry skin. Their lips become white, and they have fever. Then we guess that they have malaria*. – Itinerant drug vendors

*In this area, there’s a specific season for malaria patients. During the summer season, there are few patients the entire day. During the winter and rainy season there are many patients that come to the shop*. – Medical drug representative

*The one that I should give the test to is someone who has had malaria before. If he or she has had malaria before and now they have fever, I must give the test*. – Medical drug representative

#### Relationship of RDT use to perceived context of malaria: “there’s no more malaria”

This selective use of RDTs for only suspected malaria may in part be due to the prevailing belief among many informal providers that malaria incidence and prevalence is very low. Many informal providers believed that there was little or no malaria in the area.

*We used to have many malaria fever cases in the region, but no longer. We used to have a lot of malaria before*. – General retail seller

*When I came here there was a lot of malaria cases – not many – but at least three cases a month. Now there’s no more malaria. I hear about one or two people a year…It’s not that malaria is eradicated, but it’s rare.* – Itinerant drug vendor

Several informal providers attributed the progress on decreasing malaria to the numerous efforts made by non-profit organizations in recent years.

*Here, people have experienced malaria before. Later, after* [international non-governmental organization] *came in, it disappeared because* [international non-governmental organization] *fights for malaria*. – Itinerant drug vendor seller

*Two to three years ago this village was famous for malaria, and the groups* [health] *gave mosquito nets and talked, so malaria is very low*. – General retail seller

#### Use of RDTs for all fever cases: differences of motivations by provider type

While the majority of informal providers only used RDTs if they suspected malaria, a small number of informal providers reported they would use it for all fever cases, as seen with this itinerant drug vendor who emphasized the benefit to the patient

*I didn’t find anyone that I suspected had malaria* [previously], *but now that I have the RDT, I give the test whether it’s necessary or not if I find a fever case. If I do that, it’s beneficial for the patients. If it’s not, it’s useless. So, if I see a fever case, I always give a blood test.* – Itinerant drug vendor

One medical drug representative knew that s/he administered RDTs more frequently than needed in order to alleviate the patient’s doubts.

*If they are willing to take the test, I will give the test. If you think that I should give all those patients blood tests, I don’t think so because* [it’s] *based on my diagnosis of their symptoms. I end up giving it to them because they are willing to help them alleviate their doubts. Actually, not all of them need testing. I give more tests than I should because the patients want to alleviate their doubts.* – Medical drug representative

For this general retail seller, s/he administered RDTs because s/he knew that was the only way to confirm malaria.

*You can’t be sure if you don’t take the test. It’s better if you take the test”.* – General retail seller

Of the small number of informal providers who reported using RDTs for all fever cases, the motivations for use appear to vary by provider type. General retail sellers appear to be less comfortable relying on clinical judgment to assess suspected malaria and administer RDTs for all fever cases in order to help their clinical decision-making.

### Adherence to RDT result

#### Positive RDT result

The majority of results from RDTs administered were negative, although of those that were positive, providers recall a *P. falciparum* diagnosis more frequently than *P. vivax*. There was variability in how informal providers interpreted results, and many providers did not know how to distinguish between *P. falciparum* and *P. vivax* based on the results. Some providers said one line means no malaria but two lines means malaria, while others said the middle line means malaria and the third line is severe malaria.

Informal provider treatment knowledge was greater for *P. falciparum* compared to *P. vivax.* Supa Arte was the most common drug prescribed for *P. falciparum*; however for serious cases, a few informal providers were scared to treat and would refer patients to the hospital. A few informal providers also did not know what drug to prescribe. If the test result was positive for *P. vivax*, informal providers were unclear which drug was best to prescribe. The drugs mentioned by informal providers for treating *P. vivax* included a number of drugs, including ACT, artemisinin monotherapy and antibiotics. As one general retail seller said, *“There is no clear drug for* P. vivax*”.*

Typically if the test result showed *P. falciparum,* informal providers prescribed Supa Arte and for *P. vivax,* informal providers referred patients to the hospital.

*“If the* [RDT] *show in* P. vivax*, there is malaria and these drugs* [Supa Arte] *cannot be given for this type and the person needs to be referred to the clinic or hospital. If it is in* P. falci*parum, there is also malaria and these drugs* [Supa Arte] *can be given. If I find both types, I do not know what it is so I must refer this patient to the hospital.”* – Medical drug representative

#### Negative RDT result

Many informal providers said that after a negative RDT result they would treat the patient for common cold or flu. Informal providers mentioned an array of medicines they would prescribe, most commonly giving antibiotics. Other drugs they would give include ACT, artemisinin monotherapy and vitamins. A few informal providers said they would conduct other tests (blood pressure), refer patients to a hospital for further testing, or prescribe nothing since they did not have the right treatment. Several informal providers understood not to prescribe anti-malarials if the RDT result showed no malaria.

*If there’s no malaria, you don’t need to give any drugs. These drugs* [Supa Arte] *are only for people who have the disease*. – General retail seller

*I wouldn’t give anti-malarials to him. It’s not suitable for the patient if they don’t have malaria. So, if it’s just a fever, I will just give common drugs*. – Itinerant drug vendor

Several providers also gave examples of not prescribing anti-malarials for the last time a patient tested negative.

*I tested the child, but no malaria was found and I gave no anti-malarial drugs*. – General retail seller

*“I did the test. Even she said she has had history but the blood test showed the red line in the ‘C’ control, not in ‘*P. falciparum’ *and ‘*P. vivax’*. So I dared not give the drugs. I did not give any drugs to her. The fever is high but the result did not show positive”* – Medical drug representative

However, quite a few informal providers also gave examples of when the RDT was negative but they still gave anti-malarials, either because the patient did not get better, patient demand, or the informal provider believed the patient had malaria despite the negative RDT result.

*Another patient was also like this. He said he had fever for one week, high and intermittent for regular time. So I did the test but it showed in the ‘C’ control, not in ‘*P. falciparum’ *and ‘*P. vivax’*. He said she had malaria really and please give him anti-malarial drug. So I gave the green card* [Supa Arte] *for adult drugs…One week later, he came back to me and I asked how was your fever so he said it was relieved.* – Medical drug representative

*All were negative, just one line. I didn’t give malaria drugs at the time, only normal drugs. The patient didn’t get better. The next day, I gave quinine and artesunate. Only then did he get better*. – Medical drug representative

*Sometimes patients take the drugs – Supa Arte – based on their own decision. Because this is the shop, I have to sell it to them.* – General retail seller

Prescribing anti-malarials even after a negative RDT was more common with general retail sellers and medical drug representative.

#### Informal provider recommendation

Informal providers gave a number of recommendations for improving their experiences with RDTs and to support uptake among patients. In particular, they suggested improved training on specific areas, undertaking demand creation activities, and ensuring quality RDTs and consistent supply. This study also identified a number of knowledge gaps in provider training including treatment options for *P. falciparum vs P. vivax*. Additionally, many providers were unsure of what to prescribe for negative RDT results.

The most common recommendation by informal providers was to undertake demand creation activities. One general retail seller said, *“If the patients doesn’t accept this* [RDT], *we can’t do anything*”. This theme was echoed by many informal providers who discussed the importance of patient acceptability for use of the RDT. Many informal providers discussed increasing patient knowledge regarding malaria, including general malaria knowledge, when to get tested with a RDT, and where patients can get the test. Informal providers recommended a number of community health and marketing strategies, including using posters, TV, billboards, and information leaflets to help increase knowledge.

*Advertise it. To get awareness from most people, you must put some posters and billboards in the crowded places like the markets. Some watch the TV but others don’t. I also stick the posters in the shop, but I think this poster is a little small*. – Medical drug representative

One informal provider gave a specific recommendation to work closely with communities and leaders in the villages.

*Like I said before, discussing with village heads maybe in the schools or village meetings. When there are many villages together, you don’t need to recruit by yourself. On those occasions, you can educate them all on malaria prevention, treatment, availability of malaria treatment, places where it’s available, don’t be afraid of malaria. Other promotional items to help remind people about malaria and items that can be given to people to help remind people.* – Itinerant drug vendor

Finally, informal providers also stressed the importance of ensuring a consistent and reliable supply of RDTs.

*I think it’s best if the RDTs are available all the time. Although we use it infrequently, we should have it when we need it. Availability is important*. – General retail store

## Discussion

The informal private sector makes up a significant portion of malaria care in Myanmar, yet little is known about provider preferences and acceptability of RDTs and malaria treatment. This study demonstrated high acceptability of using RDTs. Informal providers felt the RDT was easy to use and they experienced minimal challenges. This finding reflects results from past studies in other contexts [10,11,21-25]. This study also identified several motivations for use, including increased provider empowerment to diagnose malaria and improved provider-patient relationships. Provider empowerment, in particular, was cited as a common reason for wanting to use RDTs as it improved their ability to make more informed diagnostic decisions.

This study found that the majority of informal providers used RDTs only for patients they suspected had malaria. Informal providers suspected malaria for patients who presented with a specific type of fever: fever with chills, fever that returns, or fever that is regular. Informal providers also suspected malaria for particular types of patients: those who had returned from Thailand or north, those who had worked in the field (rubber, gold, mines, migrant workers), or those who had malaria previously. Informal providers’ perception of suspected malaria for particular types of patients may be accurate given that artemisinin resistance has emerged along the Thailand and Myanmar border in the past decade [26] and that malaria prevalence is more common in certain populations, such as migrant or field workers [27].

Most informal providers believed malaria incidence and prevalence were very low in their communities, and that malaria was decreasing significantly due to recent prevention efforts by various organizations. This is in stark contrast to existing reports of high transmission and endemicity, particularly in the Mon and Shan State [2]. However, more recent monitoring and evaluation data suggests that only 8% of febrile cases tested for malaria actually had malaria [28]. Despite recent declines in transmission, Myanmar continues to account for the majority of malaria-related morbidity and mortality in the region, and feasibility challenges will further detract from a goal of elimination [29]. Providers’ perceptions that malaria has been eliminated in their communities combined with targeted RDT use may further exacerbate these challenges.

Although most informal providers did not prescribe anti-malarials for a negative RDT result, a few did, in particular general retail sellers and medical drug representatives, which raises a number of clinical concerns. Some informal providers also struggled with interpreting the RDT correctly, as well as knowing what to prescribe for positive results, especially for *P. vivax*. While the use of RDTs is feasible and acceptable by informal providers in Myanmar, there is a need to continue support and training for informal providers on treatment knowledge and adherence to RDT results. In particular, a focus on what to do for a negative RDT result is needed in future studies. This study found that many informal providers treated for the common cold or flu and prescribed an array of medicines for negative RDT results. This is corroborated by other studies that also found high prevalence of ineffective drug use, particularly antibiotics, after the introduction of RDTs [3,30]. This study demonstrated a higher capacity among itinerant drug vendors for RDT result adherence compared to other types of informal providers. Interventions focused on improving diagnosis and treatment of patients should consider the type of informal provider involved, and particularly training on when to use antibiotics.

As in any study, there are limitations to these results. First, the study is a small, qualitative study and therefore may not be generalizable to other areas of Myanmar or contexts. Future studies should validate these findings in other settings in Myanmar. Second, this study was conducted in the context of a larger community, randomized intervention of provider incentives to increase RDTs. The intervention included providing informal providers with subsidized RDTs and either information, education and counselling or a financial incentive in the form of one free RDT for every four RDTs purchased. This might specifically impact findings that provider motivations were less financially driven, and more altruistic-driven. However, other findings in Myanmar have also found altruistic motivations among private providers [31]. Providers were also purposefully sampled to include a balance of each intervention arm. More detailed information about the larger study is described elsewhere [20].

Despite these limitations, this study highlights a number of recommendations and future research priorities. First, informal providers need further specific training on procedures for a negative RDT and cases of *P. vivax*. Second, raising community level awareness of malaria symptoms and specifically about RDTs is crucial, given that these providers respond to the needs of people within their communities. Engaging with community leadership is an important strategy to building trust and improving relationships between public health programmes and communities. Moreover, due to perceptions that malaria is eliminated in their communities, providers should also be trained on when to use RDTs and on the general understanding of malaria transmission and prevalence. Finally, ensuring adequate RDT and drug supplies will be important for containment and treatment of malaria in Myanmar. Both public and private sector should engage with one another to ensure adequate supplies of commodities and that supplies are consistently available to providers.

## Conclusion

This study found that introducing RDTs to informal providers in Myanmar was highly acceptable, resulting in improved provider empowerment and patient-provider relationships. Informal providers faced challenges with adequate facility infrastructure to use and dispose RDTs and lack of provider knowledge around malaria testing and treatment. Providers also had challenges with interpreting RDT results. This suggests that refresher trainings would be helpful for future interventions with these types of providers. Perceived low prevalence of malaria also influenced use of RDTs. This study informs future interventions on RDT use in Myanmar, including specific topics to cover in trainings and considerations for working with certain types of providers.

## Abbreviations

ACT: Artemisinin-based combination therapy
PSI/M: Population Services International Myanmar
RDTs: Rapid diagnostic tests
MARC: Myanmar Artemisinin Resistance Containment
